# High Occurrence of *Staphylococcus aureus* Isolated from Fitness Equipment from Selected Gymnasiums

**DOI:** 10.1155/2018/4592830

**Published:** 2018-08-28

**Authors:** Lesley Maurice Bilung, Ahmad Syatir Tahar, Rosdi Kira, Aina Ariffah Mohd Rozali, Kasing Apun

**Affiliations:** Faculty of Resource Science and Technology, Universiti Malaysia Sarawak, Jalan Datuk Mohammad Musa, 94300 Kota Samarahan, Sarawak, Malaysia

## Abstract

**Introduction:**

*Staphylococcus aureus* is a leading cause of cutaneous bacterial infection involving community.

**Methods:**

In this study, a total of 42 swab samples were collected from the surface of various fitness equipment such as back machines, exercise mats, dip stations, dumbbells, and treadmills. Identification of the bacterial isolates was conducted using biochemical tests and further analysed molecularly using the PCR method targeting *nuc* gene (270 bp). The *nuc* gene encodes for the thermonuclease enzyme, a virulent factor of *S. aureus*.

**Results:**

The findings showed 31 out of 42 swab samples (73.81%) were positive with *S. aureus*.

**Conclusion:**

This study showed that gymnasium equipment is a potential reservoir for *S. aureus* and might play an important role in transmitting the pathogen to humans.

**Objective:**

This study was undertaken to assess the presence of *S. aureus* on the surface of fitness equipment from selected gymnasiums in Kuching and Kota Samarahan, Sarawak (Malaysia).

## 1. Introduction

*Staphylococcus aureus* is a Gram-positive and nonmotile cocci bacterium that is most common in soft tissue and skin infection [1]. Within the genus, there are 39 valid species. Animal and human pathogenic species include *S. aureus*, *S. intermedius*, *S. delphini*, *S. hyicus*, *S. schleiferi* subsp. *coagulans*, *S. pseudintermedius*, *S. equorum*, *S. xylosus*, *S. carnosus*, *S. simulans*, *S. saprophyticus*, *S. succinus*, *S. warneri*, *S. vitulinus*, *S. pasteuri*, *S. epidermidis*, and *S. lentus* [2]. *S. aureus* is a normal microflora of the skin, nose, and mucous membrane of humans where colonisation is more common than infection. Skin infection can occur if the cutaneous barrier is disrupted or damaged [3]. Any individuals that have been colonised by the bacteria are susceptible to any consequential infections and worse among patients of type 1 diabetes, intravenous drug users, patients undergoing haemodialysis, surgical patients, and patients with the acquired immunodeficiency syndrome (AIDS) [4].

The spectrum of clinical manifestations can be from minor skin infections to life-threatening illnesses. Skin infections include pimples, impetigo, boils (furuncles), cellulitis, folliculitis, carbuncles, scalded skin syndrome, and abscesses, while life-threatening illnesses include pneumonia, meningitis, osteomyelitis, endocarditis, toxic shock syndrome (TSS), bacteremia, and sepsis [5]. Procurement of skin infection is predominantly via skin contact with infected surfaces. The common high-risk groups are nursery children, patients in hospitals, prisoners, athletes, and military personnel [6, 7].

There are two trends of *S. aureus* infection. First is the increase of cases in community-acquired (CA) and hospital-acquired (HA) infection as they increased the use of intravascular devices. The second trend is highly selective antibiotic resistance which increases the infections caused by methicillin-resistant *Staphylococcus aureus* (MRSA) [4]. MRSA is an emerging strain of *S. aureus* that is resistant to several antibiotics acquired by *mecA*, a methicillin-resistant gene [6]. The strain was firstly discovered by Barrett et al. [8] at Boston City Hospital, nine years upon methicillin was introduced in the treatment of penicillin-resistant *Staphylococcus aureus*. MRSA produces similar clinical conditions as the MSSA infection but requires more aggressive antimicrobial therapy since it is highly resistant to many antibiotics. MRSA infections are related to football, fencing, rugby, wrestling, cross-country, soccer, volleyball, basketball, and weight lifting [9]. In Malaysia, the first case of infection was reported in the 1970s [10].

Up to the present time, no published studies on *S. aureus* from gymnasiums, sport facilities, or even athletes have been conducted in Malaysia. However, several studies on the presence of *S. aureus* have been reported on football teams [11–15], basketball teams [14], rugby teams [16], weight lifters [9] and wrestling teams [17]. Despite the high occurrence of *S. aureus* in sport facilities, studies taking place in Malaysia are still lacking. This study was conducted to assess the presence of *S. aureus* on the surface of fitness equipment from the selected gymnasiums in Kuching and Kota Samarahan, Sarawak (Malaysia).

## 2. Materials and Methods

### 2.1. Sample Collection

**Figure 1:**
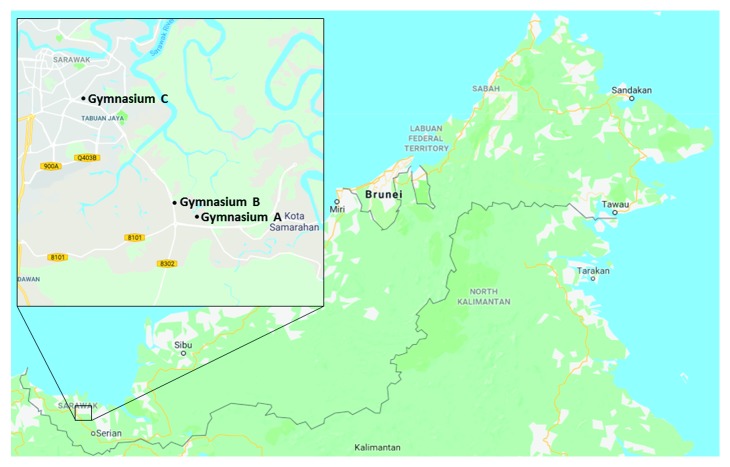
A map of gymnasiums involved in this study. Gymnasiums A and B were located in Kota Samarahan, while gymnasium C was located at BDC in Kuching.

A total of 3 selected gymnasiums located in Kota Samarahan and Kuching were identified as displayed in Figure 1, and sampling was conducted within the period of July 2016–January 2017. A total of 14 swab samples were collected from each gymnasium and analysed by following the swab method as in William et al. [18]. By using sterile cotton swabs, surfaces of sport equipment such as dumbbells, kettlebells, exercise mats, treadmills, dip stations, and back machines were swabbed and stored in individual sterile universal bottles containing phosphate-buffered saline. Afterwards, the bottles were placed in an ice box and transported to the Molecular Microbiology Laboratory, Universiti Malaysia Sarawak, for further analysis.

### 2.2. Biochemical Tests

**Figure 2:**
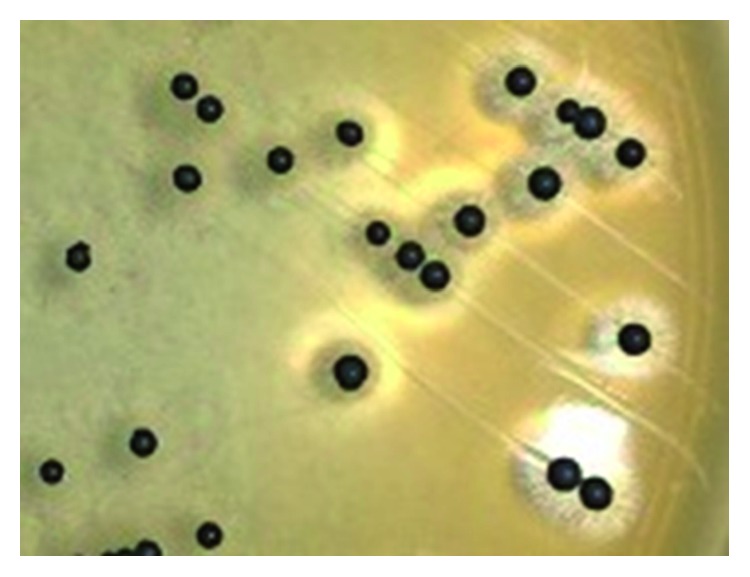
Formation of black colonies surrounded with clear zones indicating *Staphylococcus aureus* on the Baird-Parker agar.

Detection of *S. aureus* using the biochemical test was in accordance with the method of William et al. [18]. All swab samples were streaked on the selective Baird-Parker agar and incubated at 37°C for 24 hr. Presumptive growth of *S. aureus* was detected based on the formation of black and clear zones of colonies on the agar plates as shown in Figure 2. Three colonies from each plate were picked and used in Gram staining and catalase test, a test that can distinguish *S. aureus* from other catalase-negative staphylococci.

### 2.3. DNA Extraction

DNA extraction was performed using the boiled cell method by Fitrianda et al. [19] with a little modification. The isolates were grown in the Luria-Bertani agar at 37°C for 24 hr. Subsequently, 1 ml of the culture was centrifuged at 12,000 rpm for 2 min. The pellets were resuspended in 500 *μ*l sterile distilled water and followed with vortexing. The suspension was boiled at 100°C for 2 min and promptly chilled on ice for 15 min. The suspension was centrifuged at 12,000 rpm for 5 min, and the supernatant was used for PCR analysis.

### 2.4. Specific Polymerase Chain Reaction (PCR)

Molecular analysis using specific PCR was performed in accordance with the method of Ali et al. [20]. A primer pair targeting *nuc* gene was used (*nuc*-F: 5′-GCGATTGATGGTGATACGGTT-3′; nuc-R: 5′-AGCCAAGCCTTGAACGAACTAAAGC-3′). 25 *µ*l of the reaction mixture consisted of 1x Green GoTaq Flexi buffer (Promega, United States), 1.5 mM of MgCl_2_, 0.5 mM of each dNTP, 10 mM of each primer, 1.25 U of GoTaq DNA Polymerase (Promega, United States), and 20–30 ng of DNA template. The PCR reaction was carried out with initial denaturation at 95°C for 5 min, 37 cycles each of denaturation at 95°C for 30 seconds, annealing at 55°C for 30 seconds, and extension at 72°C for 1 min, and a cycle of final extension at 72°C for 10 min. Positive and negative controls were included. Amplified fragment analysis was done in 1.5% agarose gel electrophoresis. A 100 bp molecular weight DNA ladder was included (Thermo Fisher Scientific, United States). The gel was viewed under a UV transilluminator (Maestrogen, Taiwan).

## 3. Results

### 3.1. Biochemical Test

**Figure 3:**
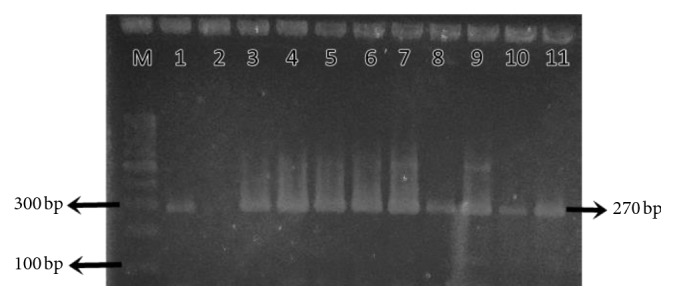
Representative of agarose gel electrophoresis of PCR amplification products from seven isolates of *S. aureus*. Lane M: 100 bp molecular weight DNA ladder (Thermo Fisher Scientific, United States); lane 1: positive control of *S. aureus*; lane 2: negative control; lanes 3 to 11: sample isolates from the gymnasiums.

Black colonies on the Baird-Parker agar indicated presence of the *S. aureus* morphological characteristic. As shown in Table 1, presumptive *S. aureus* detection using the Baird-Parker agar, Gram staining, and catalase test revealed gymnasium A had the highest number of positive isolates (14/14; 100%), followed by gymnasium B (13/14; 92.86%) and gymnasium C (4/14; 28.57%). These numbers were in tandem with confirmation using the PCR assay targeting *nuc* gene (270 bp). A representative of agarose gel electrophoresis is shown in Figure 3.

## 4. Discussion

High occurrence of *S. aureus* was detected from gymnasiums A and B, compared to gymnasium C. It was noted that gymnasium C was open for 24 hours and had twice-a-day disinfection (i.e., morning and night), whereas gymnasium A and B were open only during working hours and had once-a-day disinfection before closing. Based on CDC [21], disinfectants effective against MSSA can be used for MRSA. A list of many approved disinfectants is available on the US EPA [22] website where the public can check for application. Household chlorine bleach may also be used, but the persons must pay attention to the instruction, dilution, and exposure time for ensuring an effective disinfection [23]. Bacteria on sport equipment can persist if topical disinfectant is applied that cannot penetrate deeper into interstices of the surface [24].

The current finding obtained a high number of samples positive for *S. aureus* (73.8%). The potential of MRSA could contaminate gymnasium equipment and would be equal to that of MSSA [25] which should receive attention by the public and owners. Circulation of *S. aureus* in the gymnasium area was also reported by Cohen [26] where three weight lifters from the University of Houston were infected with MRSA, who eventually recovered after several treatments which were incision and drainage, sensitivity-directed antibiotic therapy, topical mupirocin, and cleansing with an antibacterial agent (povidone-iodine or chlorhexidine). Other pathogens can also be isolated from gymnasium equipment such as in a study by Goldhammer et al. [24] that found diphtheroids, Gram-positive cocci, *Bacillus* species, coagulase-negative staphylococci, *Micrococcus* species, and culturable virus from two fitness centres.

Many studies have been conducted to assess any risk factors associated with the infection among athletes. For instance, a study by Kazakova et al. [27] found that methicillin-resistant *Staphylococcus aureus* (MRSA) infection was frequent among footballers (35/84; 42%), where they reported the risks were worsened by several factors, for example: the games were conducted on artificial turf instead grass, hand sanitisers were limited, alcohol-based medication was not given during wound care, sharing towels, players did not take shower before using whirlpools, and training and therapy equipment was not disinfected periodically. Nevertheless, these factors may vary depending on the athlete behaviours and facility.

Gymnasium trainers play a crucial role in controlling the spread of MRSA within the facility. Based on the study by Rihn et al. [1], athletic trainers and therapists must be alert for MRSA infection among athletes, be able to identify skin lesions susceptible to MRSA infection, adhere to the knowledge that culturing infectious lesions is important in making accurate diagnosis and subsequent antibiotic treatment, cover infectious lesions with a clean and dry dressing until they are healed, encourage athletes to cover any risky areas from injury and cover skin injury with a clean, dry dressing, educate athletes on CA-MRSA illness and hygienic practices, and produce reports to local infectious disease specialists or CDC on incidences of CA-MRSA infection. This current study detected *S. aureus* utilising PCR primers targeting *nuc* gene, a unique marker used widely in *S. aureus* detection in food and clinical samples. The gene encodes for the thermonuclease enzyme in the bacteria, a virulent factor that hydrolyses DNA and RNA of infected cells [28]. Utilisation of the gene allows rapid detection of *S. aureus* [20].

Limitations faced in this study were small sample size and that this study is not specifically focused on MRSA detection which would be significant to determine any potential level of *S. aureus*-causing skin infections. Several gymnasiums in Kuching and Kota Samarahan were reluctant to permit sample collection being carried out on their equipment due to some issues (i.e., trust and concern over hygienic practice) among the customers that they may be affected. The small sample size in this study is not representative of the whole gymnasiums in Kuching and Kota Samarahan. However, it provides preliminary assessment of high occurrence of *S. aureus* on equipment from the selected gymnasiums.

## 5. Conclusion

This study revealed a relatively high prevalence of *Staphylococcus aureus* on the surface of the gymnasium equipment from the selected gymnasiums in Kuching and Kota Samarahan. As this is the first one of such reports in Malaysia, more studies are important to provide more insight into MRSA strains from the local gymnasiums. Although no cases have been reported regarding *S. aureus* infections within gymnasiums in the country, the public and gymnasium owners need to practice good hygiene and sanitisation to avoid any infections which potentially cause an outbreak in the future.

## Figures and Tables

**Table 1:** Biochemical tests and PCR assay on all the samples from gymnasiums A, B, and C.

Gymnasium	Location	Formation of black colonies growing on the Baird-Parker agar^a^	Gram staining	Positive reaction to the catalase test^a^	PCR assay targeting *nuc* gene^a^
A	Kota Samarahan	14/14	14 Gram positive	14/14	14/14
B	Kota Samarahan	14/14	13 Gram positive	13/14	13/14
C	BDC in Kuching	11/14	4 Gram positive	4/14	4/14
Total	—	39/42 (92.9%)	31 Gram positive (73.8%)	31/42 (73.8%)	31/42 (73.8%)

^a^Number of positive samples per total number of samples.

## Data Availability

The data used to support the findings of this study are included within the article.
